# Detection of African swine fever virus in pigs in Southwest Nigeria

**DOI:** 10.14202/vetworld.2021.1840-1845

**Published:** 2021-07-19

**Authors:** Emmanuel Jolaoluwa Awosanya, Babasola Oluseyi Olugasa, Fufa Ido Gimba, Mohd Yusoff Sabri, Gabriel Adetunji Ogundipe

**Affiliations:** 1 Department of Veterinary Public Health and Preventive Medicine, Faculty of Veterinary Medicine, University of Ibadan, Ibadan, Nigeria; 2 Department of Veterinary Pathology and Microbiology, Faculty of Veterinary Medicine, Universiti Putra Malaysia, 43400 UPM, Serdang, Selangor, Malaysia

**Keywords:** African swine fever virus, enzootic, evolution, phylogeny, sequencing, Southwest Nigeria

## Abstract

**Background and Aim:**

Nigeria experienced repeated outbreaks of African swine fever (ASF) in pig herds between 1997 and 2005 in the southwest region of the country. ASF is believed to currently be enzootic in this region. The status of enzootic transmission of ASF virus strain to pigs is; however, unknown. Twenty-three genotypes of the ASF virus based on the *p72* gene are found across Africa. This study aimed to identify the current circulating field strain(s) of the ASF virus in Southwest Nigeria and characterized evolutionary trends.

**Materials and Methods:**

DNA samples were extracted from 144 pooled blood samples obtained from 2012 to 2013 following the manufacturer’s instructions. DNA was used for conventional polymerase chain reaction using primers targeting the *p72* gene and amplified products sequenced with Sanger’s sequencing. Sequences were analyzed for homology and phylogenetic relationships.

**Results:**

Eleven of 144 samples (7.6%) showed bands at 950 bp. A new field strain of ASF virus of genotype I that shared ancestry with ASF virus strains or isolates from Spain and Brazil was identified among pig herds. The new strain differs phylogenetically in amino acid composition compared with previously identified ASF virus field strains.

**Conclusion:**

The currently circulating field strain of ASF virus suggests a mutation responsible for decreased morbidity and mortality recorded in sporadic cases.

## Introduction

African swine fever (ASF) is a highly contagious disease of domestic pigs caused by a DNA arbovirus in the family, Asfarviridae. It crosses both local and international borders and may trigger fatal outbreaks, especially in naïve pig herds. ASF has a devastating socioeconomic impact on pig farmers’ livelihood globally and especially in developing countries [1,2]. ASF virus (ASFV) lacks serotypes because it does not induce neutralizing antibodies; however, virus strains are identified genetically using sequencing at the C-terminus of the p72 protein. This process has identified 23 ASF genotypes [3-5] in Europe, and Central and South America. Genotype I is implicated in previous outbreaks. Genotype II is reported in Europe, the Caucasus, and recently in China [6]. This highly virulent strain caused 100% mortality in pigs and wild boars in Georgia, Caucasus region in 2007 [7]. In West Africa, only genotype 1 is reported in outbreaks, while all 23 genotypes are reported in South and East Africa [4,5].

The first unconfirmed outbreak of ASF in Nigeria was reported in 1973 at a commercial pig farm in Ogun state, one of the present study locations. Confirmed ASF outbreaks began in Ogun in 1997, 1998, and 2001 and spread to other parts of the country; the episode in 2001 in Oyo state was devastating with morbidity and case fatality of 100% [8]. The ASF strain implicated in the 1997/1998 outbreaks was also implicated in the 2001 outbreak – ASFV Isolate 98/ASF/Ng [9,10]. Some viral isolates of Nigerian origin, including virus major capsid protein *VP72* gene, partial cds; Nig-2 p72; Nig/2/98 p72, are deposited in GenBank [3,9,11]. These field isolates are associated with mortality of 50-100% [10]. The disease has apparently become enzootic, with sporadic cases still being reported. The most recent reported ASF outbreak in Southwest Nigeria occurred in 2020 [12].

However, since the time, the status of ASFV in Nigeria became enzootic, circulating strain(s) have not been characterized. Such characterization will aid understanding of ASF spread and maintenance in domestic pig populations. This study screened apparently healthy pigs to identify circulating field strain(s) of ASFV in the enzootic state between 2012 and 2013 in Southwest Nigeria and identify evolutionary trends.

## Materials and Methods

### Ethical approval and Informed consent

Ethical approval was obtained from the University of Ibadan Animal Care and Use Research Ethics Committee (UI-ACUREC) – UI-ACUREC/App/2015/063. The study was carried out in compliance with the guidelines of the UI-ACUREC. Informed consent was obtained from all participating pig farm owners.

### Study period and location

The study was conducted from November 2012 to June 2015 in all six states in Southwest Nigeria.

### Sample preparation and nucleic acid extraction

Blood samples were collected from 657 healthy pigs from 144 pig farms in six states (Ekiti, Lagos, Ondo, Ogun, Osun, and Oyo) in Southwest Nigeria. Pigs were selected randomly at each farm. Pig farms practiced both intensive and semi-intensive “farrow to finish” rearing systems without any history of vaccination against common diseases. Samples were pooled by farm. DNA was extracted using a commercial kit (Wizard^®^ Genomic DNA Purification Kit [Quick Protocol/Instruction for use of products A1120, A1123, A1125 and A1620]) following the manufacturer’s instructions. DNA was rehydrated in 70 μL of DNA rehydration solution and stored at −80°C until use.

### PCR amplification and sequencing

Amplification used conventional ­polymerase chain reaction (PCR) [13] with primers based on the conserved region of the nucleocapsid protein isolated from Nigerian ASF virus, AF159503 [9]: ASFV-capsid-F-sequence 5′-TCTTGCGATCTGGATTAAGCTGCGC-3′ (ASFV-F: forward primer); ASFV-capsid-R-sequence 5′-CACAAGATCAGCCGTAGTGATAG-3′ (ASFV-R: reverse primer) generate an amplicon of 895 bp. The PCR master mix (25 μL reaction) used sterile distilled water (17.375 μL), 10× PCR buffer (2.5 μL) (Thermo Scientific^®^, USA), magnesium chloride 25 mM (2 μL) (Thermo Scientific^®^), PCR nucleoside mix (dNTP mix) 10 mM of each nucleotide (0.5 μL) (Thermo Scientific^®^), primer ASFV-F, 20 pmol/μL (0.25 μL), primer ASFV-R, 20 pmol/μL (0.25 μL) (Integrated DNA Technologies IDT^®^, Singapore), Dream Taq Green DNA Polymerase 5 U/mL (0.125 μL) (Thermo Scientific^®^), and sample DNA template (2 μL). The positive reference was ASFV strain E70 (OIE reference laboratory, Spain) and the negative reference used sterile distilled water. The PCR mixture was prepared on ice in bulk for the number of samples to be assayed with two extra samples in a sterile 1.5 μL microcentrifuge tube. Twenty-three microliters of the PCR reaction mixture were placed in 200 μL PCR tubes before the addition of 2 μL of sample DNA template or positive or negative control to make up the 25 μL reaction. The reactions were placed in a thermal cycler with a heated lid (Model PTC-150 MiniCycler™). The following thermal cycling program [13] was used with a slight modification in annealing temperature from 62°C to 52°C based on the melting temperature of the primers: Initial denaturation at 95°C for 10 min (one cycle); denaturation at 95°C for 15 s; annealing at 52°C for 30 s and extension at 72°C for 30 s (40 cycles); and final extension at 72°C for 7 min (one cycle). Amplified products were separated by electrophoresis in 1.7% agarose gels in tris-acetate-EDTA (TAE) buffer and examined under ultraviolet light. The 1.7% agarose gel was prepared by mixing 0.51 g of agarose in 30 μL of TAE (1×) and 5.1 μL of gel red. The mixture was heated in a microwave for 1 min, then allowed to cool before being poured into the PCR gel plate with a 12-well comb. A 100 bp ladder (6 μL) was added to one lane on each side of the gel. The gel was run at a constant voltage of 90 volts and 400 amperes for 45 min; the amplified PCR products were sent to 1^st^ Base Sequencing^®^, Singapore, for purification and sequencing. Sanger sequencing was done using ABI PRISM 3730 × l Genetic Analyzer, Applied Biosystem^®^, USA. The kit that was used for the sequencing was BigDye^®^ Terminator v3.1 Cycle Sequencing kit, Applied Biosystem, USA.

### Sequence analysis and phylogeny

Sequences were analyzed with BioEdit® Sequence Alignment Editor Version 7.1.9. The Basic Local Alignment Search Tool was used to identify similarities with reference strains/isolates from GenBank. The ClustalW process in MEGA6 [14] was used to align sequences before constructing the phylogenetic tree. All gaps and missing data in the sequence alignment (in the form of dashes [-]) were deleted. The genotype of the sequence from this study was compared with other known genotypes from GenBank. Initial trees for the heuristic search were obtained automatically by applying neighbor-joining and BioNJ algorithms to a matrix of pairwise distances estimated using maximum composite likelihood. The topology with a superior log-likelihood value was then selected. The neighbor-joining method [15] was used to determine the evolutionary history of the study sequence. The optimal tree with the sum of branch length was selected automatically. The percentage of replicate trees in which the associated taxa clustered together in the bootstrap test (1000 replicates) is shown next to the branches. Evolutionary distances were computed using the Kimura 2-parameter method [16] and presented in units of the number of base substitutions per site. Codon positions included were 1^st^ + 2^nd^ + 3^rd^ + noncoding. All positions containing gaps and missing data were deleted. Evolutionary analyses were conducted in MEGA6 [14]. Two hundred and fifty-seven nucleotide positions were identified in the final p72 datasets. A summary of all ASFV strains/isolates used in the study is presented in Table-1 [3,4,9,17-24].

**Table-1:** Summary of African swine fever viruses analyzed in this study.

S/N virus designation	Country	Year	GenBank accession no.	Reference
ASFV_AEJ_ID132	Nigeria	2015	-	This study
46/Ca/08_(NI)	Italy	2010	FR668416	[17]
ASFV_VP72_Madagascar_(II)	Madagascar	2002	KP144287	[18]
TAN/12/Iringa_(NI)	Tanzania	2012	KF834193	[19]
NIG-2_(I)	Nigeria	1998	AF504884	[3]
11/Og/04_(NI)	Italy	2010	FR668403	[17]
Brazil80_(I)	Brazil	2014	KJ526367	[20]
CAM/2006/ 1_(I)	Cameroon	2006	KC662377	[21]
ASFV_S691-88_(I)	Russia	2014	KJ671549	[22]
NIG/2/98_(I)	Nigeria	1998	AF159503	[9]
ASFV-PPA_(1)	Spain	2014	KJ526362	[23]
ZIM/92/1	Zimbabwe	2005	DQ250119	[4]
MAL2011/ 4_(NI)	Malawi	2011	JX524217	[24]
TOGO/98_(I)	Togo	2001	AF449489	[3]
MOZ/1/98_(II)	Mozambique	2000	AF270705	[3]
BOT/1/99_(III)	Botswana	2002	AF504886	[3]
TENGANI/60(V)	Malawi	2000	AF301541	[3]
UGA/1/95_(IX)	Uganda	2001	AF449475	[3]

### Statistical analysis

Descriptive statistics, such as frequencies and percentages, were calculated. Maximum-likelihood methods were used in sequence analyses.

## Results

### PCR results

**Plate-1:**
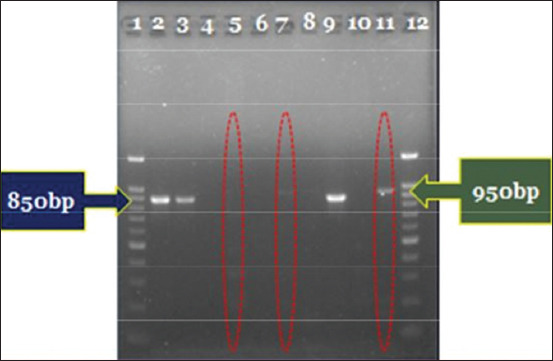
Gel picture of the electrophoresis separation of the amplified PCR products showing bands at 950 bp.

Eleven of 144 blood samples pooled by the farm (7.6%) were positive, as indicated by amplicons of 950 bp (Plate-1); no amplicons corresponded exactly with the expected size of 895 bp.

### Sequence and phylogenetic analyses

**Figure-1:**
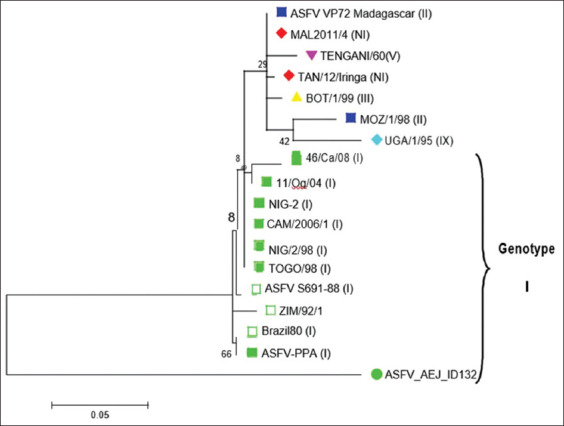
Phylogenetic tree indicating the genotype relationship of ASF virus strains based on p72 gene (ML). A total of 257 nucleotide positions were analyzed after the removal of gaps and ambiguous residues. A maximum-likelihood tree based on the Kimura 2-parameter model was used to infer the evolutionary history. The tree with the highest log likelihood (−791.6255) is shown. Branch lengths indicate the number of substitutions per site. The ASF strain from this study was indicated with a green dot.

**Figure-2:**
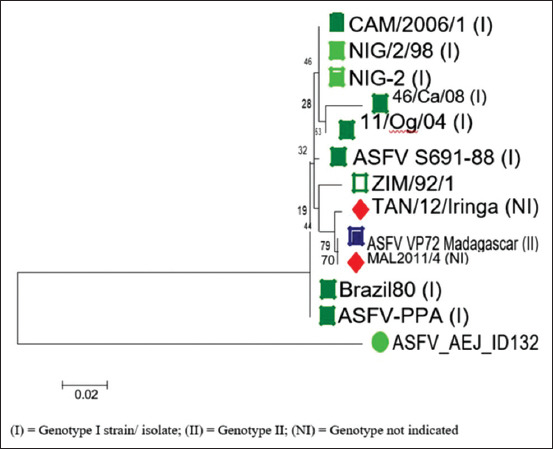
Phylogenetic tree indicating the evolutionary history of ASF virus strains based on p72 gene (NJ). A total of 257 nucleotide positions were analyzed after the removal of gaps and ambiguous residues. One thousand bootstrap repetitions were performed to improve clade confidence. Neighbor-joining tree computed using Kimura 2-parameter method with units indicating the number of base substitutions per site. The ASF virus strain from this study was indicated with a green dot.

Nucleotide sequences of samples with 950 bp amplicons showed an alignment score of 139. Identity was 81% with Expect (E) values close to zero. The occurrence of similar matches by chance thus approached zero. The query cover was 69% with most reference strains/isolates from GenBank irrespective of genotype. The local ASFV strain (ASFV_AEJ_ID132) displayed 78.5% nucleotide identity with reference strains/isolates, mostly within genotype I. This genotype includes reference strains/isolates from Nigeria (Accession number AF159503); Cameroon (Accession number KC662377); Spain (Accession number KJ526362); Brazil (Accession number KJ526367); and Russia (Accession number KJ671549) (Table-2). The evolutionary divergence between the *p72* gene of reference ASFV strains or isolates and the study ASFV strain (ASFV_AEJ_ID132) showed pairwise values ranging from 0.28 to 0.30 (Table-3). The phylogenetic tree distance of isolates/strains placed ASFV_AEJ_ID132 in genotype I (Figure-1). The ASFV_AEJ_ID132 strain shares its ancestry with ASFV strains or isolates from Spain and Brazil (Figure-2). Differences were observed in amino acid composition of strain ASFV_AEJ_ID132 and other ASFV strains or isolates from GenBank. Percentage identity with reference amino acids from GenBank was between 22.9% and 33.3%.

**Table-2:** Percent homology of study strain (ASFV_AEJ_ID132) with African swine fever virus reference strains/isolates from the GenBank.

S/N virus designation	Country	GenBank accession no.	Percent identity with ASFV_AEJ_ID132 (%)
ASFV_AEJ_ID132	Nigeria	-	100.0
Brazil80_(I)	Brazil	KJ526367	78.5
CAM/2006/1_(I)	Cameroon	KC662377	78.5
ASFV_S691-88_(I)	Russia	KJ671549	78.5
NIG/2/98_(I)	Nigeria	AF159503	78.5
ASFV-PPA_(1)	Spain	KJ526362	78.5
ASFV_VP72_Madagascar_(II)	Madagascar	KP144287	78.1
TAN/12/Iringa_(NI)	Tanzania	KF834193	78.1
NIG-2_(I)	Nigeria	AF504884	78.1
11/Og/04_(NI)	Italy	FR668403	78.1
MAL2011/4_(NI)	Malawi	JX524217	78.1
46/Ca/08_(NI)	Italy	FR668416	77.7
ZIM/92/1	Zimbabwe	DQ250119	77.3

**Table-3:** Estimates of evolutionary divergence between *p72* gene of ASFV_132 strain. The number of base substitutions per site between sequences is shown.

Strains	1	2	3	4	5	6	7	8	9	10	11	12	13
ASFV_AEJ_ID132		0.04	0.04	0.04	0.04	0.04	0.04	0.04	0.04	0.04	0.04	0.04	0.04
strain_46/Ca/08_(NI)	0.30		0.01	0.01	0.01	0.01	0.01	0.01	0.01	0.01	0.01	0.01	0.01
ASFV_VP72_Madagascar_(II)	0.29	0.03		0.00	0.01	0.01	0.01	0.01	0.01	0.01	0.01	0.01	0.00
TAN/12/Iringa_(NI)	0.29	0.04	0.00		0.01	0.01	0.01	0.01	0.01	0.01	0.01	0.01	0.00
NIG-2_(I)	0.29	0.02	0.01	0.02		0.00	0.00	0.00	0.00	0.00	0.00	0.01	0.01
strain_11/Og/04_(NI)	0.29	0.02	0.02	0.02	0.00		0.01	0.00	0.01	0.00	0.01	0.01	0.01
Brazil80_(I)	0.28	0.02	0.02	0.02	0.00	0.01		0.00	0.00	0.00	0.00	0.01	0.01
CAM/2006/1_(I)	0.29	0.02	0.01	0.02	0.00	0.00	0.00		0.00	0.00	0.00	0.01	0.01
ASFV_S691-88_(I)	0.29	0.02	0.02	0.02	0.00	0.01	0.00	0.00		0.00	0.00	0.01	0.01
NIG/2/98_(I)	0.29	0.02	0.01	0.02	0.00	0.00	0.00	0.00	0.00		0.00	0.01	0.01
strain_PPA_(I)	0.28	0.02	0.02	0.02	0.00	0.01	0.00	0.00	0.00	0.00		0.01	0.01
ZIM/92/1_(NI)	0.30	0.04	0.02	0.02	0.02	0.02	0.01	0.02	0.02	0.02	0.01		0.01
MAL2011_4_(NI)	0.29	0.03	0.00	0.00	0.01	0.02	0.02	0.01	0.02	0.01	0.02	0.02	

## Discussion

A new enzootic field strain (ASFV_AEJ_ID132) of the ASF virus, type I genotype, was identified among healthy pigs. The nucleotide sequence of the new strain (ASFV_AEJ_ID132) is identical to earlier ASFV isolates/strains deposited in GenBank [9] (accession no. AF159503 [78.5%]) and Bastos *et al*. [3] (accession no. AF504884 [78.1%]). These strains were associated with previous ASF outbreaks in Nigeria. It also shares close similarities with strains from Cameroon, Brazil, Spain, and Russia. The ASFV strain involved in the ASF outbreak of 1998 shared 97.2% homology with a previously sequenced ASF isolate from Spain [9]. The evolution of ASF viruses in Southwest Nigeria reflects modifications in both the pathogenicity and virulence with associated decreases in morbidity and mortality [8]. Such changes often characterize enzootic variants.

The genetic sequence variation of the new field strain suggests a high degree of variability which is sometimes due to silent mutation [25]. A genotype I strain identical to ASF virus that has caused outbreaks in neighboring Benin (ASFV Benin 97/1) was previously reported to persist among pigs in Nigeria [26]. The ASFV isolate implicated in the 1998 outbreak in Nigeria (NIG/2/98) also shares ancestry with the ASFV strain from Spain (accession no.: KJ526362). These associations are consistent with trade and importation of foreign swine or artificial insemination with semen from countries, such as Sardinia and South Africa, where the virus is enzootic [27,28]. Transboundary movement of infected animals is implicated in the spread of ASF outbreaks in South Africa using a two-step genetic characterization approach [4,5,28]. The ASFV in the recent outbreak in Southwest Nigeria [12] has not been characterized.

There are significant differences in the amino acid composition and identity of the new field strain compared to some other members of genotype I. The dissimilarity in the amino acid composition could be due to amino acid substitution [29] or mutation. Changes may result from vaccination of pig herds with classical swine fever vaccines by some medium- and large-scale pig farmers in Southwest Nigeria, as observed in earlier epizootic [1]. The marked difference in amino acid composition between the study strains and other Nigerian strains previously identified by Odemuyiwa *et al*. [9] and Bastos *et al*. [3], and some members of genotype I could account for some differences in epidemiological and clinical manifestations observed in the field in terms of the spread of the virus and decreased morbidity and mortality.

The prevalence of ASF in the study area was 7.6% (11 of 144), which is less than herd seroprevalence of 28% found by enzyme-linked immunosorbent assay (ELISA) [8]. This finding corroborates the sensitivity of the ELISA test as suitable for screening of ASF infection. The presence of ASFV DNA in study samples suggests the presence of the virus but not necessarily its active form. The active form can cause infection and spread to naïve pigs.

The study had some limitations. First, the isolation of the ASFV from the study samples failed due to deterioration arising from improper storage and a time lag of about 5 years when virus isolation was attempted. These problems hampered attempts at revalidation. Second, a delay of about 18 months between sampling collection and DNA extraction could have resulted in a complete lack or poor amplification of some sequences, despite the use of standard optimization processes, with a consequent reduction in the number of PCR amplicons produced. Despite these setbacks, this study suggests the possibility of a new circulating enzootic strain of ASFV.

## Conclusion

This study identified a possible new enzootic strain of ASFV belonging to genotype I circulating among pig herds in Southwest Nigeria. The virus in Southwest Nigeria appears to have undergone significant genetic mutation and shares its ancestry with strains or isolates from Spain and Brazil. The evolution of the virus in Southwest Nigeria might reflect the observed decreases in morbidity and mortality in the study areas, consistent with an enzootic strain. Federal and state governments should step up surveillance, especially for international purchases of livestock and biologicals, such as frozen semen from countries, such as Sardinia and South Africa, where enzootic strains exist. Stakeholders in the pig farming business should be educated on smart practices, such as vaccination of pigs with classical swine fever vaccine and control of the importation of semen harboring the enzootic virus, if eradication of ASFV is sought. Further studies are recommended for validation and continuing surveillance of circulating ASFV among pigs presented for slaughter and to sequence the viral strain involved in the recent outbreak in Southwest Nigeria.

## Authors’ Contributions

EJA and BOO: Conceptualization. EJA: Data acquisition. EJA, FIG, and MYS: Methodology. BOO, MYS, and GAO: Supervision. EJA: Manuscript drafting. All authors read, reviewed, and approved the final manuscript.
